# Supplementary material for: Spatiotemporal mapping of malaria prevalence in Madagascar using routine surveillance and health survey data
Source: Sci Rep. 2020 Oct 22;10:18129. doi: 10.1038/s41598-020-75189-0 (PMC7581764; doi:10.1038/s41598-020-75189-0)
Supplement: Supplementary file 1 — Supplementary Information. [file 41598_2020_75189_MOESM1_ESM.pdf]

Spatiotemporal mapping of malaria prevalence in Madagascar using routine surveillance and health survey data

Rohan Arambepola[1*], Suzanne H. Keddie[2], Emma L. Collins[1], Katherine A. Twohig[1], Punam Amratia[1], Amelia Bertozzi-Villa[1,8], Elisabeth G. Chestnutt[1], Joseph Harris[2], Justin Millar[1], Jennifer Rozier[2], Susan F. Rumisha[1], Tasmin L. Symons[1], Camilo Vargas-Ruiz[1], Mauricette Andriamananjara[5,7], Saraha Rabeherisoa[5], Arsène C. Ratsimbasoa[5,6], Rosalind E. Howes[1,4], Daniel J. Weiss[1,2,3], Peter W. Gething[1,2,3], and Ewan Cameron[1,2,3]

[1]Big Data Institute, Li Ka Shing Centre for Health Information and Discovery, University of Oxford, Oxford, United Kingdom
[2]Telethon Kids Institute, Perth Children's Hospital, Perth, Australia
[3]Curtin University, Perth, Australia
[4]Foundation for Innovative New Diagnostics, Geneva, Switzerland
[5]Programme National de Lutte contre le Paludisme, Antananarivo, Madagascar
[6]University of Fianarantsoa, Fianarantsoa, Madagascar
[7]Ministère de Santé Publique, Antananarivo, Madagascar
[8]Institute for Disease Modeling, Bellevue, WA, USA
[*]rohan.arambepola@stx.ox.ac.uk

Supplementary material

Catchment population sensitivity analysis

To assess the sensitivity of our results to our methods of estimating catchment populations, the analysis was repeated with three different catchment population estimates: NMCP estimates, catchment model estimates with higher treatment-seeking and catchment model estimates with lower-treatment seeking. In the lower treatment-seeking setting, the same logistic form was assumed but parameters were chosen such that maximum and minimum treatment-seeking proportions of 0.875 and 0 and a proportion of 0.1 at $t = 90$ minutes. In the higher treatment-seeking setting, maximum and minimum treatment-seeking proportions were 0.6 and 0.25, with a proportion of 0.3 at $t = 200$ minutes.

Figure 1 shows the incidence surfaces in these three scenarios across the four years, while Figure 2 shows the final prevalence estimates. We see that while the magnitude of incidence is significantly different between in these three scenarios, the overall spatial and temporal patterns and similar and the resulting prevalence estimates and therefore also fairly similar.

Treatment-seeking parameterisation

Figure 3 shows the relationship between treatment-seeking and travel time to nearest health facility observed in the 2013 and 2016 MISs and the relationship used in the catchment model. The shape of these relationships is similar but the treatment-seeking rates are systematically higher in the relationship used in the model to match the overall estimated treatment-seeking rates.

Monthly risk maps

Monthly maps of point prevalence estimates and interquartile ranges are shown in Figures 6 and 7. The probability of prevalence exceeding 0.15 and not exceeding 0.05 each month is shown in Figures 8 and 9.

Feature sets

Features sets found in the first step of the causal feature selection procedure are listed below. A number following a dynamic covariate indicates a time lag in months (for example, Rainfall 1 indicates the Rainfall covariates with a one month time lag). "dtw" represents distance to water and "AI" the aridity index.

- Accessibility, dtw, Rainfall 0
- Accessibility, AI, dtw, Rainfall 0
- Accessibility, dtw, LST night1, Rainfall 0
- Accessibility, AI, dtw, Slope, Rainfall 0
- Accessibility, AI, dtw, LST night1, Rainfall 0
- Accessibility, dtw, LST night 1, Rainfall0, TCB 2
- Accessibility, AI, dtw, Slope, LST night 1, Rainfall 0
- Accessibility, AI, dtw, LST night 1, Rainfall 0, TCB 2
- Accessibility, dtw, LST night 1, Rainfall 0, TCB 2, TSI Pf 2
- Accessibility, AI, dtw, Slope, LST night 1, Rainfall 0, TCB 2
- Accessibility, AI, dtw, LST night 1, Rainfall 0, TCB 2, TSI Pf 2
- Accessibility, AI, dtw, Night lights, PET, Slope, TWI, Rainfall 0
- Accessibility, AI, dtw, Slope, LST night 1, Rainfall0, TCB 2, TSI Pf 2
- Accessibility, AI, dtw, Night lights, PET, Slope, TWI, LST night 1, Rainfall 0
- Accessibility, AI, dtw, Night lights, PET, Slope, TWI, LST night 1, Rainfall 0, TCB 2
- Accessibility, AI, dtw, Night lights, PET, Slope, TWI, LST night 1, Rainfall 0, TCB 2, TSI Pf 2

Additional figures

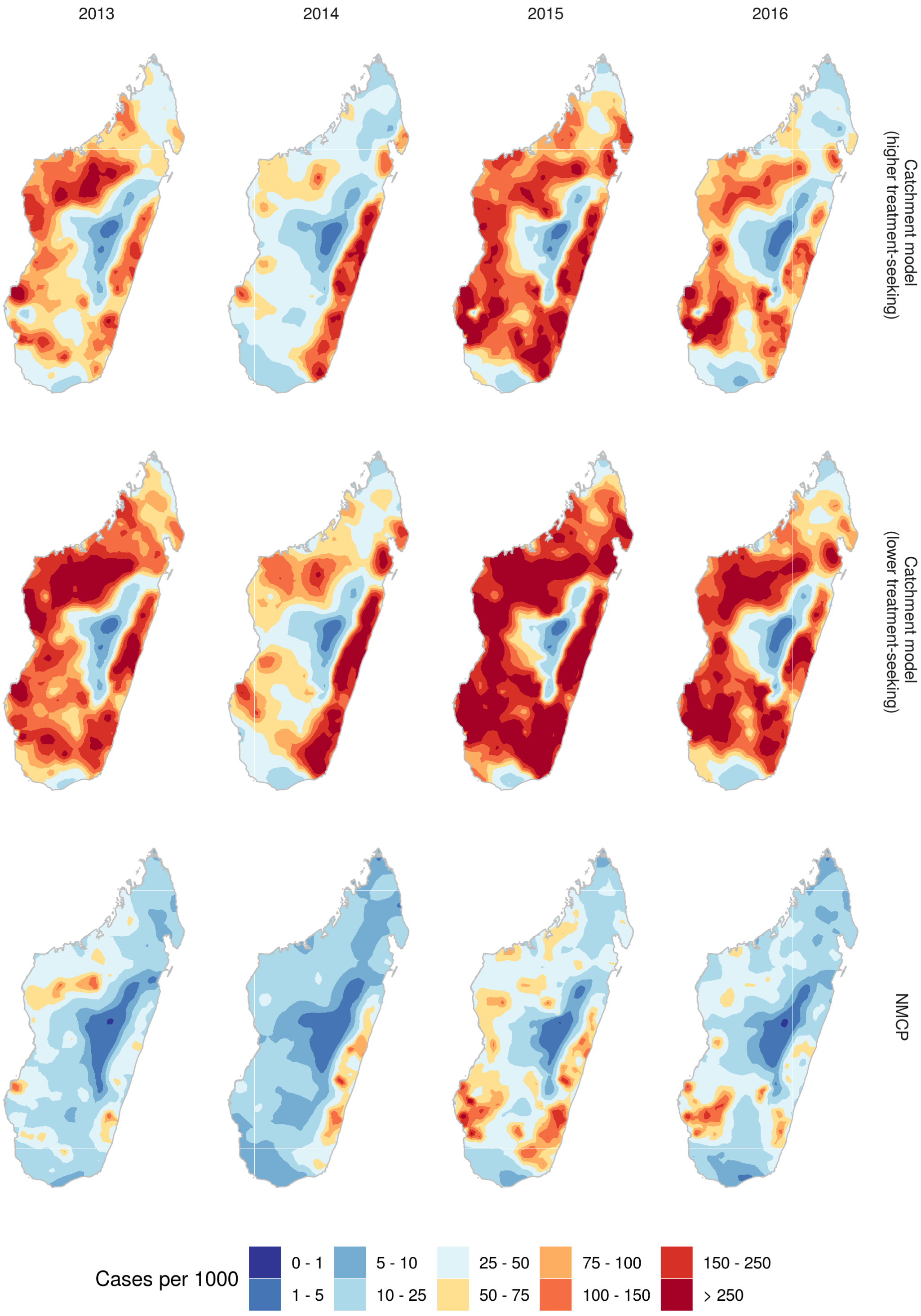

Figure 1. Annual incidence surfaces using three alternative catchment population estimates: estimates from the catchment model assuming higher treatment-seeking (top), estimates from the catchment model assuming lower treatment-seeking (middle) and estimates supplied by the NMCP (bottom).

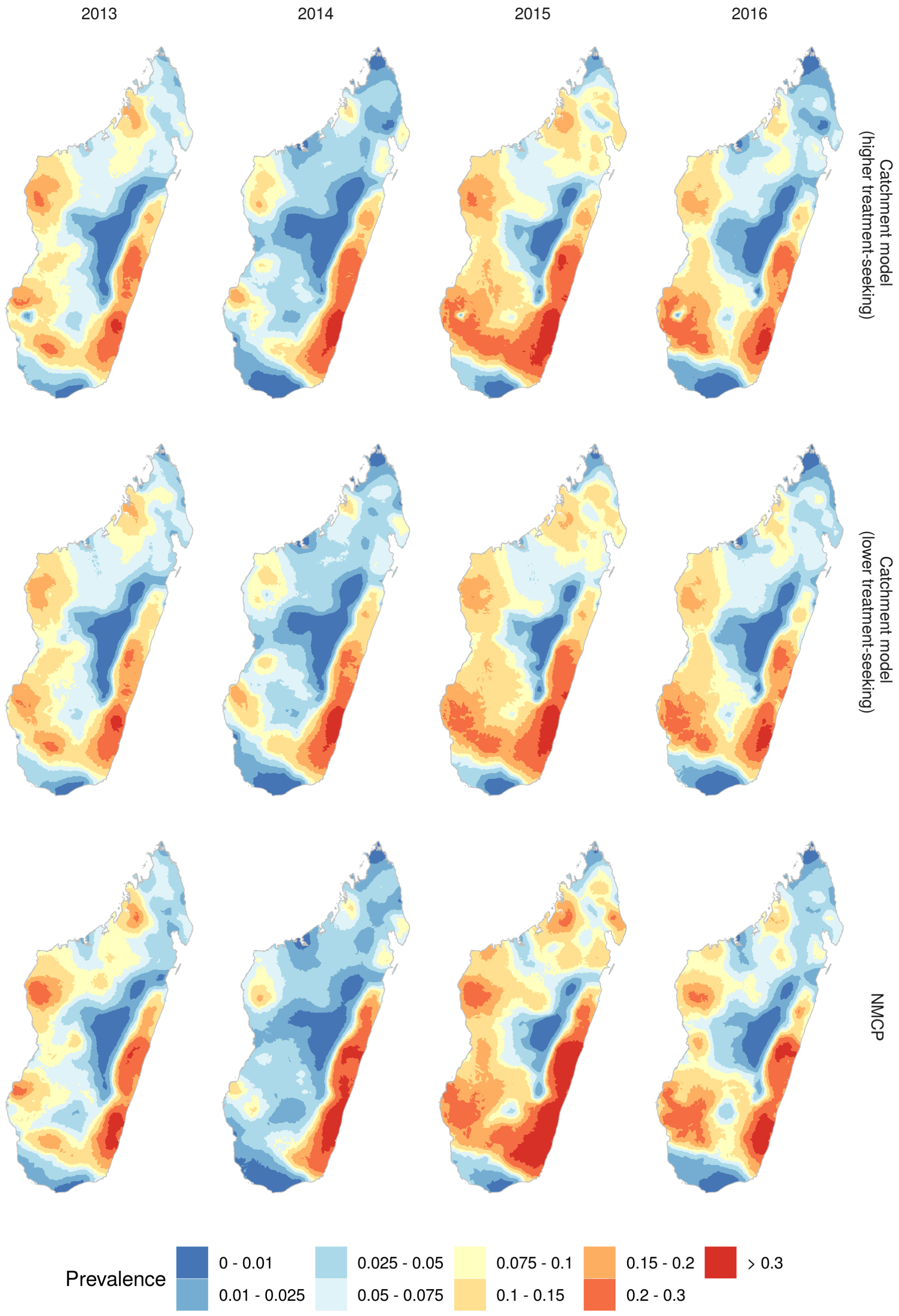

Figure 2. Annual prevalence estimates using three alternative catchment population estimates: estimates from the catchment model assuming higher treatment-seeking (top), estimates from the catchment model assuming lower treatment-seeking (middle) and estimates supplied by the NMCP (bottom).

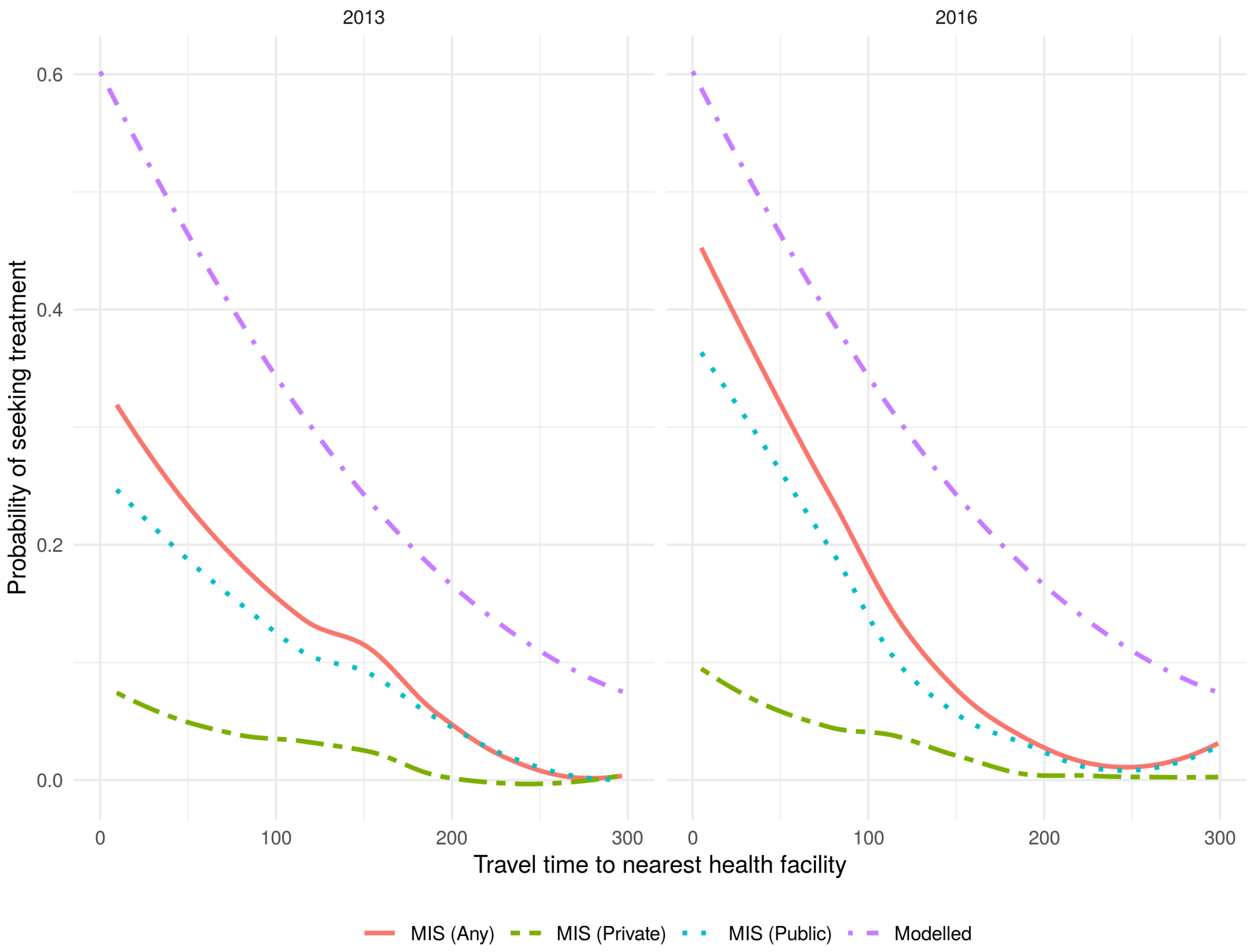

Figure 3. Locally smoothed estimates of treatment-seeking rates (at public, private and any facility) against travel time to the nearest health facility from the 2013 and 2016 MISs and the relationship used in the catchment model.

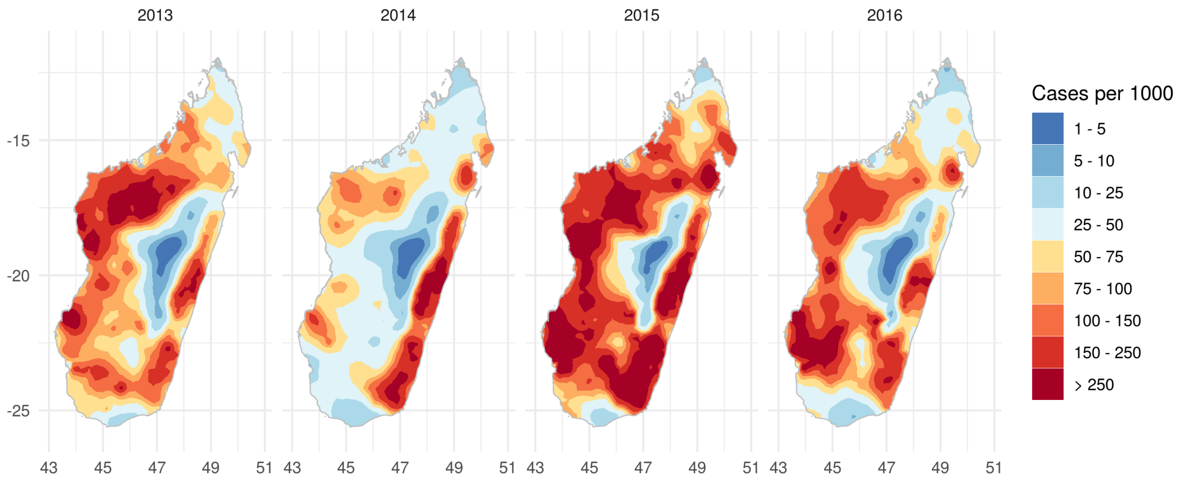

Figure 4. Incidence surfaces for all ages aggregated annually.

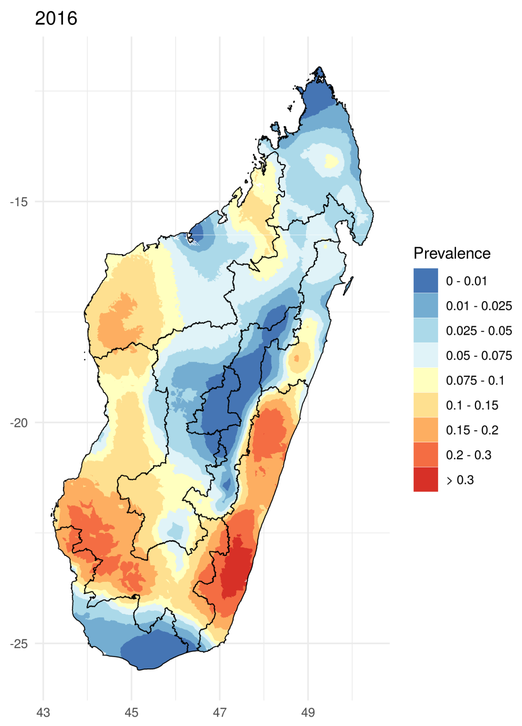

Figure 5. Prevalence estimates for 2016 with ecozones borders shown in black.

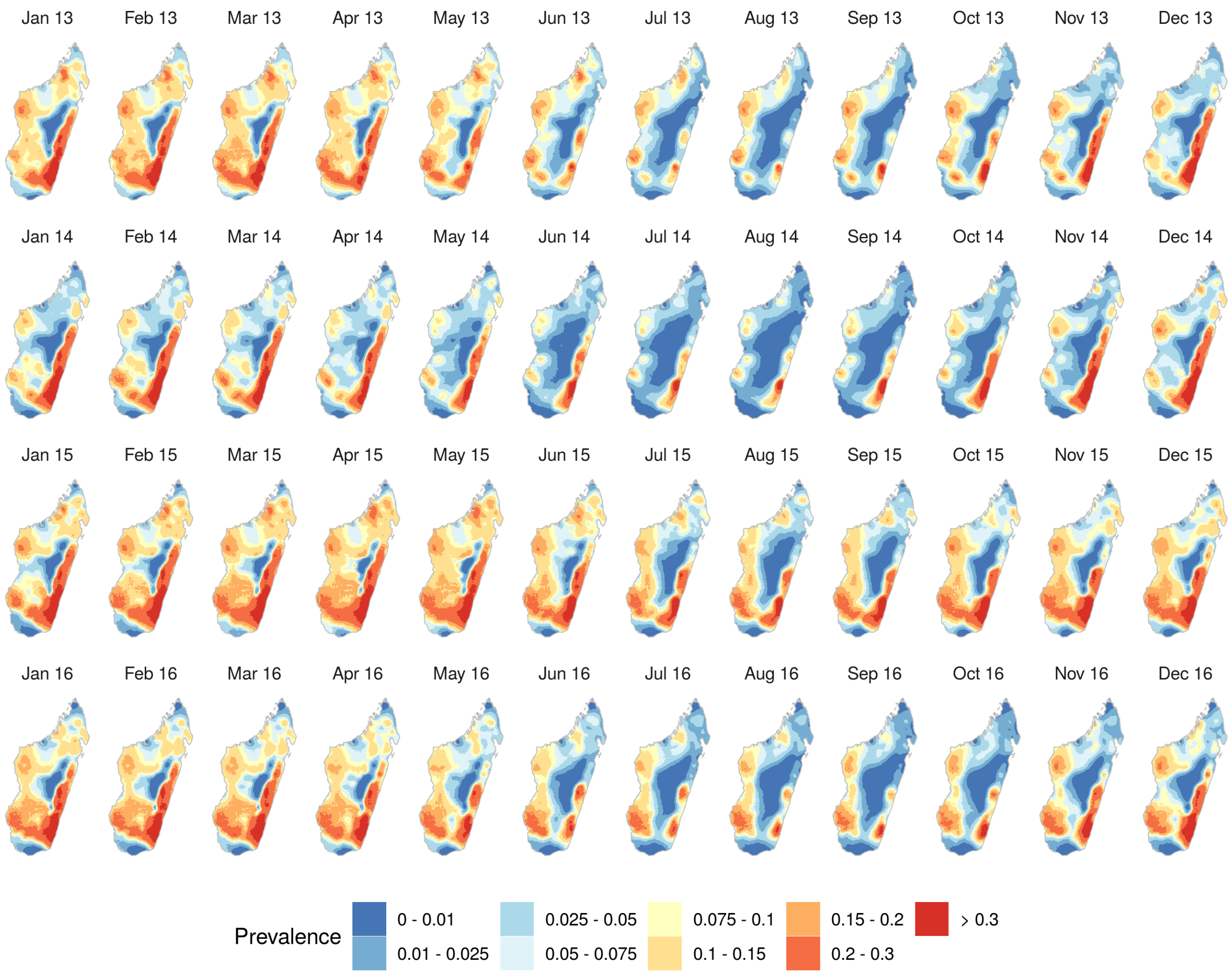

Figure 6. Monthly prevalence estimates.

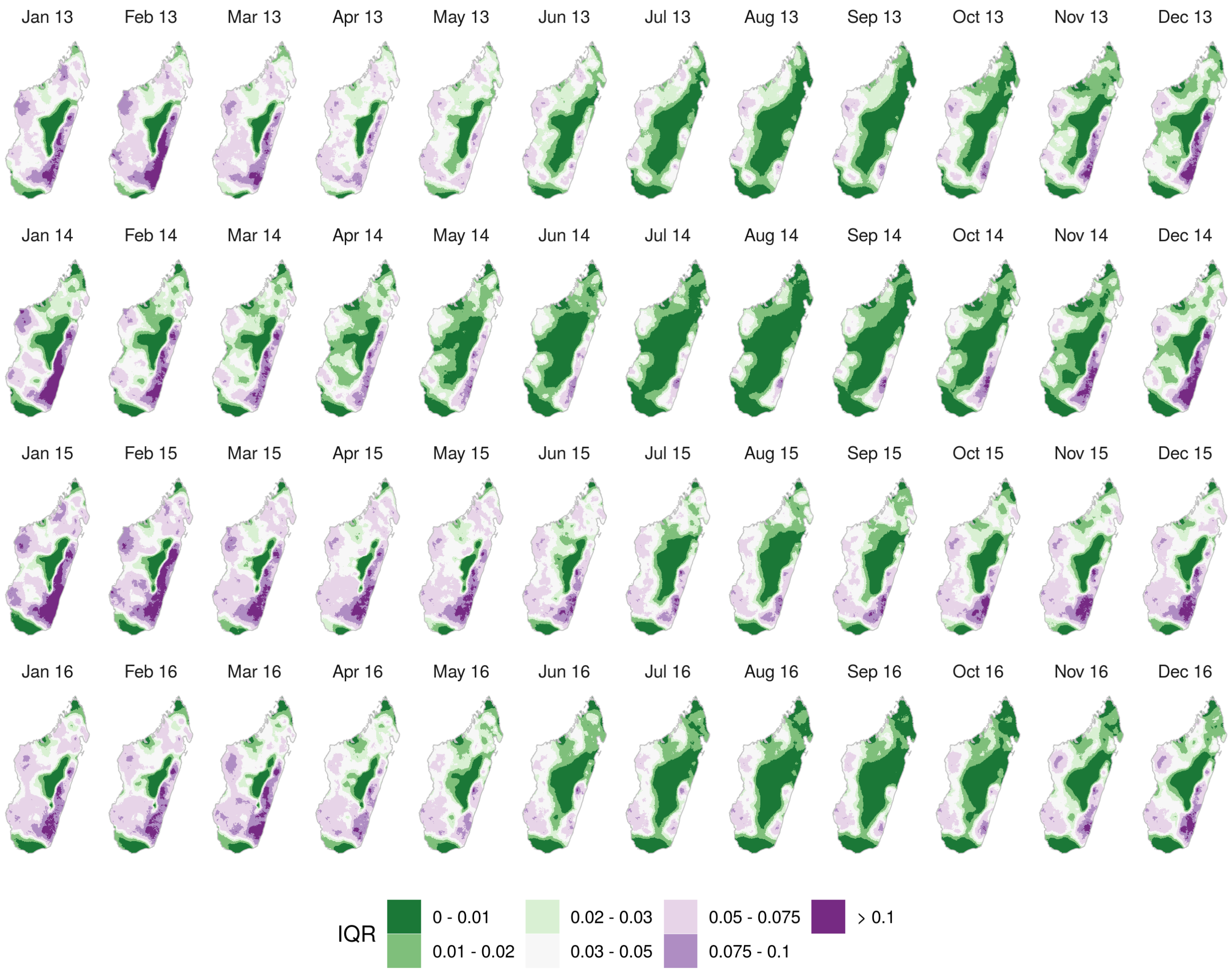

Figure 7. Monthly interquartile ranges.

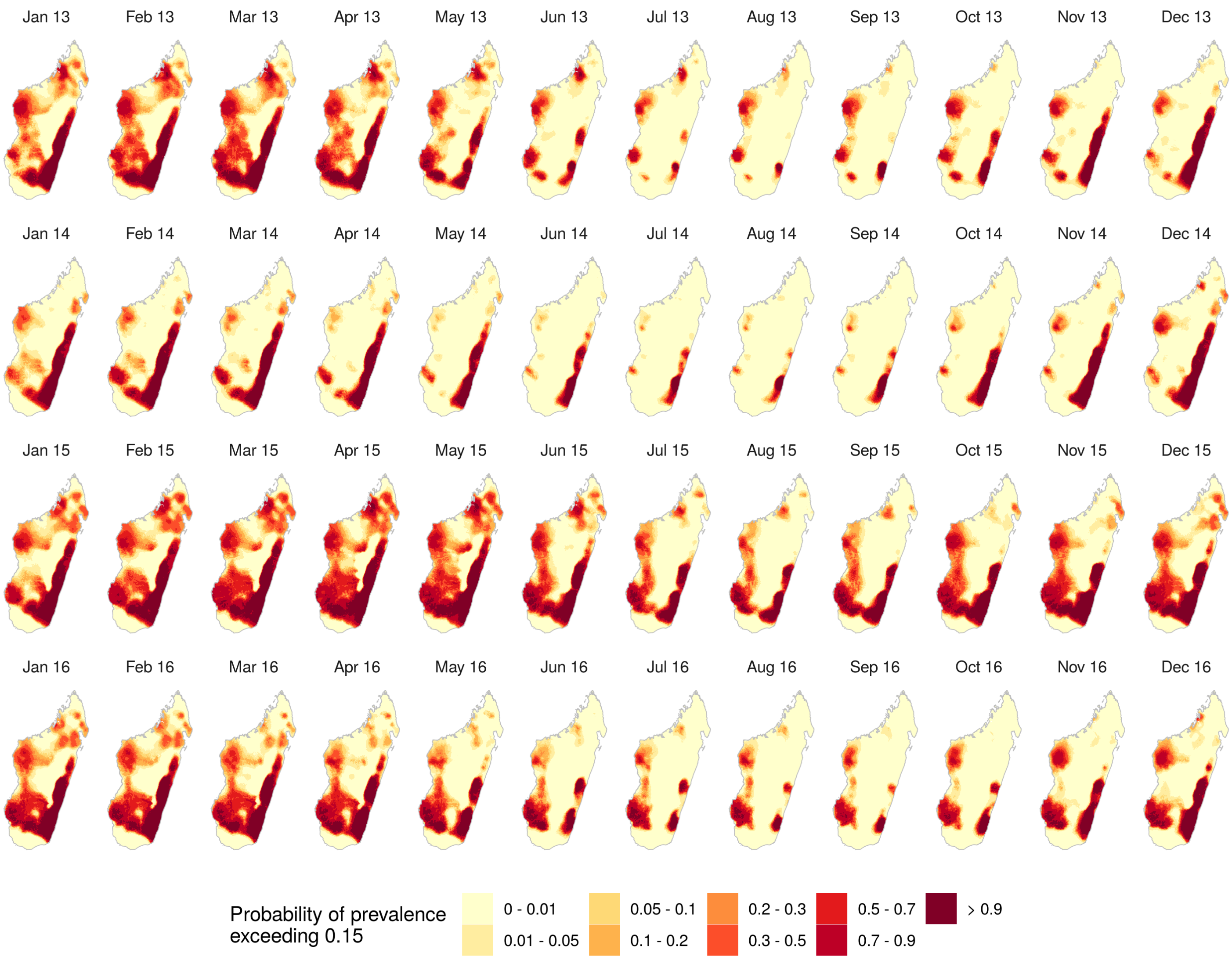

Figure 8. Probability of prevalence exceeding 0.15 each month.

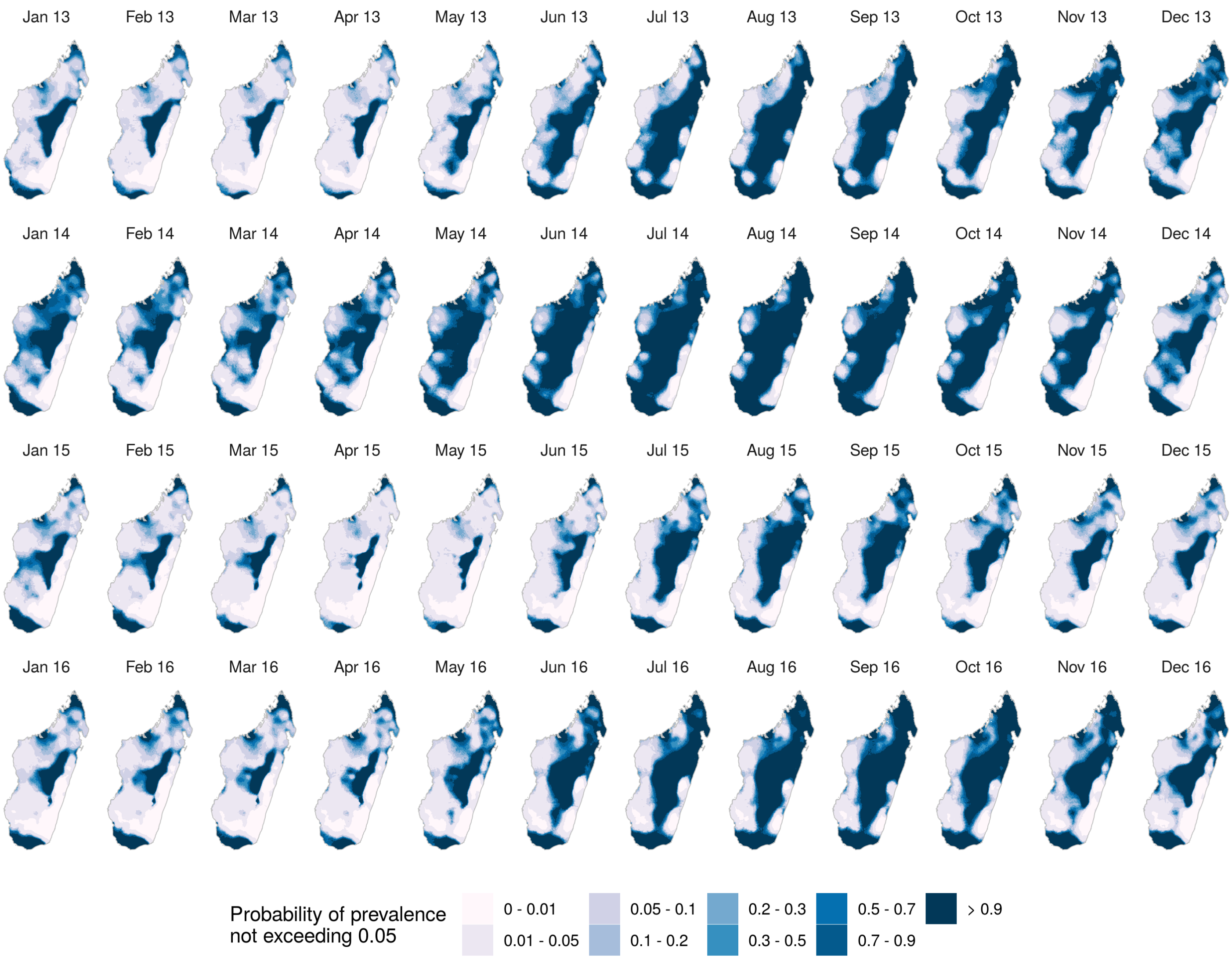

Figure 9. Probability of prevalence not exceeding 0.05 each month.
